# Retraction Note to: High expression level and nuclear localization of Sam68 are associated with progression and poor prognosis in colorectal cancer

**DOI:** 10.1186/s12876-021-01952-w

**Published:** 2021-10-11

**Authors:** Wen-Ting Liao, Jun-Ling Liu, Zheng-Gen Wang, Yan-Mei Cui, Ling Shi, Ting-Ting Li, Xiao-Hui Zhao, Xiu-Ting Chen, Yan-Qing Ding, Li-Bing Song

**Affiliations:** 1 Department of Pathology, Nanfang Hospital, Southern Medical University, Guangzhou, 510515 Guangdong People’s Republic of China; 2 State Key Laboratory of Oncology in Southern China, Department of Experimental Research, Sun Yat-Sen University Cancer Center, Guangzhou, 510060 Guangdong People’s Republic of China; 3 Department of Pathology, School of Basic Medical Sciences, Southern Medical University, Guangzhou, 510515 People’s Republic of China; 4 State Key Laboratory of Oncology in South China, Department of Medical Oncology, Sun Yat-Sen University Cancer Center, Guangzhou, 510060 Guangdong People’s Republic of China; 5 Department of Gastroenterology, The Second Affiliated Hospital, University of South China, HengYang, 421000 Hunan People’s Republic of China

## Retraction note to: BMC Gastroenterol 2013, 13:126 10.1186/1471-230X-13-126

The Editor has retracted this article after a number of concerns were raised, including:

- Overlapping panels in Figures 2 and 3
- Inaccurate description of Figure 2 parts A-C in the figure legend
- Lack of discussion of the difference between the behaviour of the cell line COLO205 compared with the other cell lines used
- Lack of methodological information relating to the 9 paired colorectal cancer tissue samples
- No description of normal cell lines used and inconsistencies in the descriptions of colorectal cell lines

The Editor no longer has confidence in the results and conclusions presented. Yan-Mei Cu agrees with this retraction. Wen-Ting Liao and Li-Bing Song disagree with this retraction. Jun-Ling Liu, Zheng-Gen Wang, Ling Shi, Ting-Ting Li, Xiao-Hui Zhao, Xiu-Ting Chen and Yan-Qing Ding have not responded to correspondence from the Editor about this retraction.

